# Interacting Learning Processes during Skill Acquisition: Learning to control with gradually changing system dynamics

**DOI:** 10.1038/s41598-017-13510-0

**Published:** 2017-10-16

**Authors:** Nicolas Ludolph, Martin A. Giese, Winfried Ilg

**Affiliations:** 1 Section Computational Sensomotorics, Department of Cognitive Neurology, Hertie Institute for Clinical Brain Research, and Centre for Integrative Neuroscience, University of Tübingen, Tübingen, Germany; 2 International Max-Planck Research School for Cognitive and Systems Neuroscience, University of Tübingen, Tübingen, Germany

## Abstract

There is increasing evidence that sensorimotor learning under real-life conditions relies on a composition of several learning processes. Nevertheless, most studies examine learning behaviour in relation to one specific learning mechanism. In this study, we examined the interaction between reward-based skill acquisition and motor adaptation to changes of object dynamics. Thirty healthy subjects, split into two groups, acquired the skill of balancing a pole on a cart in virtual reality. In one group, we gradually increased the gravity, making the task easier in the beginning and more difficult towards the end. In the second group, subjects had to acquire the skill on the maximum, most difficult gravity level. We hypothesized that the gradual increase in gravity during skill acquisition supports learning despite the necessary adjustments to changes in cart-pole dynamics. We found that the gradual group benefits from the slow increment, although overall improvement was interrupted by the changes in gravity and resulting system dynamics, which caused short-term degradations in performance and timing of actions. In conclusion, our results deliver evidence for an interaction of reward-based skill acquisition and motor adaptation processes, which indicates the importance of both processes for the development of optimized skill acquisition schedules.

## Introduction

Sensorimotor learning is a term commonly used to capture the variety of neural changes taking place (i) during the acquisition of a new motor skill or (ii) during the adaptation of movements to changed circumstances such as changes of the environment. Most extensively, motor adaptation to external perturbations has been studied over the last decades in experimental paradigms like visuomotor rotation^1,2^ and velocity-dependent force-fields^3,4^. The underlying mechanism is considered to be a re-calibration of internal forward models, which predict the sensory consequences of the motor action^5,6^. During the adaptation process, these internal forward models are re-calibrated based on a sensory prediction error^7^ which is the difference between the internal forward model prediction and the actual sensory outcome.

In recent years, a particular interest has emerged in studies which compare the sudden application of perturbations to gradually induced perturbations^8,9^, as well as the influence of reward-based feedback on the adaptation process^10–12^. In contrast to the described motor adaptation paradigms, motor skill acquisition describes the expansion of the motor repertoire when faced with completely new demands, such as learning for the first time how to ride a bicycle, monocycle or acquiring a new sports skill^13–15^.

Although studies in sports science have been examining skill acquisition for many years on a descriptive level^14,16,17^, computational studies investigating the underlying control mechanisms have mainly been restricted to simplified experimental paradigms. Examples for such paradigms are the learning of finger-tapping sequences^18^, visually-guided hand movement trajectories^15^, simplified virtual-reality versions of moving a cup^19^, bouncing a ball^20,21^ or playing skittles^22^. The focus of these studies is mainly the quantitative analysis of the execution performance and performance variability within the skill acquisition process^13^. However, processes of acquiring or adjusting internal models and specific control mechanisms like predictive control have not been the addressed in this context.

Predictive control based on forward models is suggested to play an important role in many dynamic skills like bouncing or catching a ball^17^ and performing fast goal directed movements^23–25^. More generally speaking, skilled motor behaviour is suggested to rely on accurate predictive models of both our own body and tools we interact with^26^. Indeed, studies have collected increasing evidence that the brain acquires and uses an internal model that encodes the physical properties of our limbs^27^, environment and manipulated objects^28–31^. Thus, internal forward models of new tools or objects have to be acquired during skill acquisition and have to be adjusted to changes of body dynamics during development or to external changes when the dynamics of the object change^32^.

The dynamics of external objects change for instance frequently during skill acquisition when different objects or sport devices are used as part of the training, such as different tennis rackets^33–35^. In the beginning of the learning process devices are used which are easier to handle and thus lead earlier to successful behaviour. These moments of success and resultant motivation are known to be important factors in motor learning and have been reported in several studies on motor adaptation or learning in sports^36–38^. On the other hand, at the transition to another device, the subject has to adapt the control behaviour to the dynamics of the new device within the process of skill acquisition. Thus, two learning processes, one driven by reward (skill acquisition) and the other by sensory prediction error (motor adaptation), shape the behaviour concurrently.

In this study, we want to investigate the influence of interleaved adaptation phases during skill acquisition by examining subjects’ behaviour in the virtual cart-pole balancing task while manipulating the gravity. Increments in gravity do not only change the dynamic behaviour of the system but also the difficulty to control it, allowing us to study the interaction of reward-based and error-based learning. Cart-pole balancing has been studied in the context of reinforcement learning as a benchmark for computational algorithms^39^, as model for human balance control^40–44^ and in the context of internal forward models^31,44^. We investigate the implications of gradually increasing the task complexity (i) on the skill acquisition and (ii) in terms of the need to adapt to repetitively changed cart-pole dynamics. The hypothesis under investigation is that a gradual increase in task complexity leads to improved learning due to earlier and more frequent positive reward, which is however disturbed by short interleaved adaptation phases caused by the gradually changing cart-pole dynamics.

## Results

### Experimental Design

**Figure 1:**
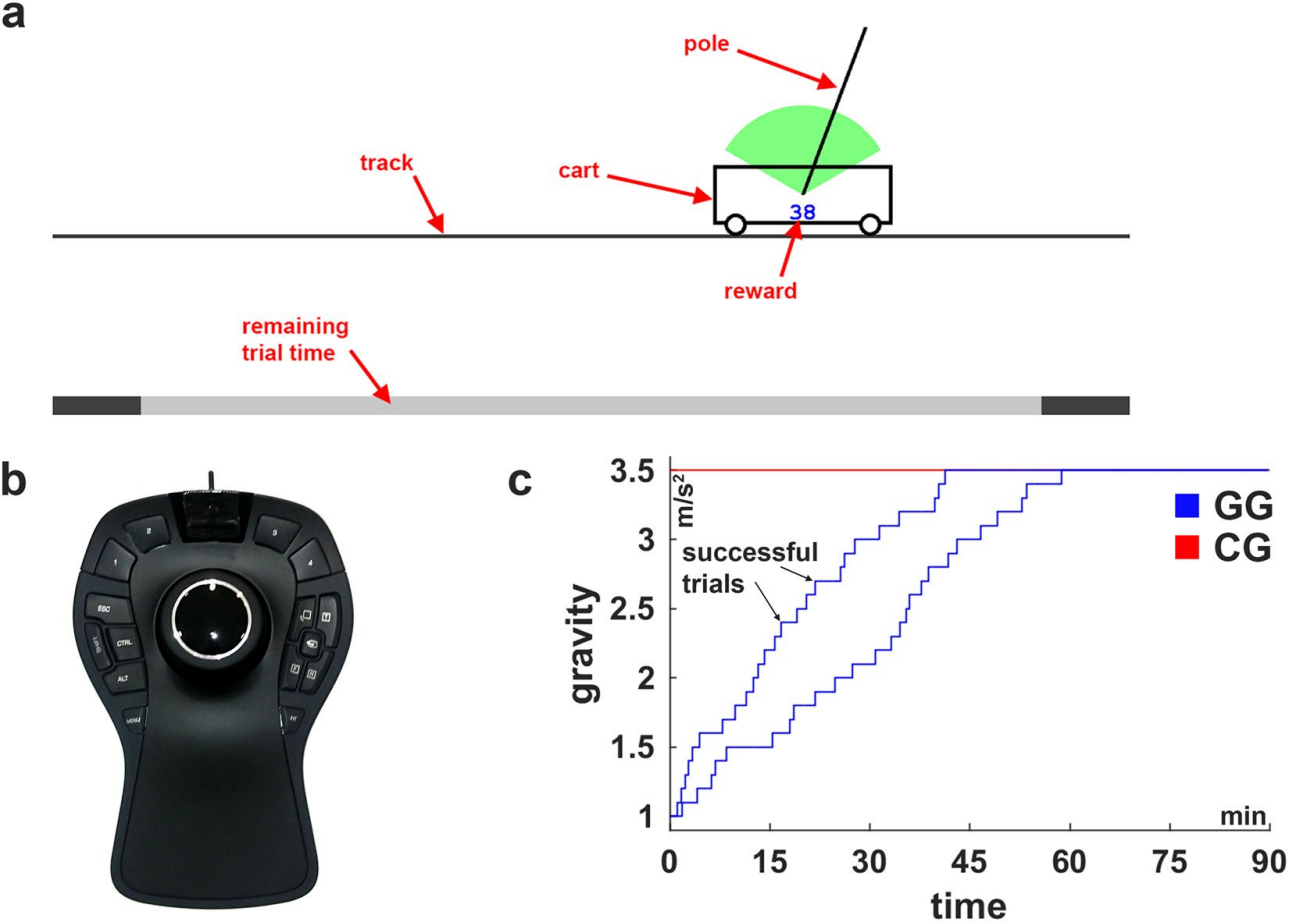
Illustrations of the cart-pole system, the used input device and experimental conditions. (**a**) Cart-pole system. (**b**) Input device. The knob of the input device can be shifted and rotated into all directions. The left-right translation was used to control the virtual force, which is applied to the cart from either side. (**c**) Experimental conditions: gradual gravity (GG) and constant gravity (CG). For the condition GG, the course of the gravity is shown for two representative subjects to illustrate the individual, performance-dependent increase. Gravity was increased after every successful balancing attempt.

Participants had to learn the control of a physically simulated cart-pole system (Fig. 1a, see Methods for details). Gravity forces the pole to rotate downwards when not being perfectly upright. Thus, in the simulation, the complexity of the control task can gradually be manipulated by adjusting the simulated gravity. Higher gravity leads to a faster falling pole and to a more complex control problem. Participants controlled the car by applying virtual forces using a haptic input device (Fig. 1b). The goal was to keep the pole upright. Specifically, the pole has to remain within the green circular segment (±60 degree, Fig. 1a) while the cart must not leave the track (±5 m). A trial is considered a successful trial if balance was maintained for 30 seconds without violating the two given constrains.

We examined two groups of subjects corresponding to different experimental conditions (Fig. 1c): (i) gradual gravity (GG), starting with a low gravitational constant of g_0_ = 1.0 m/s^2^, the virtual gravity has been increased in relation to the current performance up to the maximum of g_max_ = 3.5 m/s^2^. Specifically, the gravitational constant was increased by 0.1 m/s^2^ after every successful trial (performance-dependent increase in gravity). (ii) Constant gravity (CG), in this group the gravity has been kept constant on the maximum of g_max_ = 3.5 m/s^2^ from the beginning. Notice, that the gravitational constant was never decreased and that every subject was exposed to an individual gravity profile over the course of the experiment, due to the performance-dependent increase. Subjects in both groups interacted for 90 minutes with the cart-pole environment, while the number of trials during this time was not limited.

### Skill acquisition is facilitated in the gradual gravity condition

**Figure 2:**
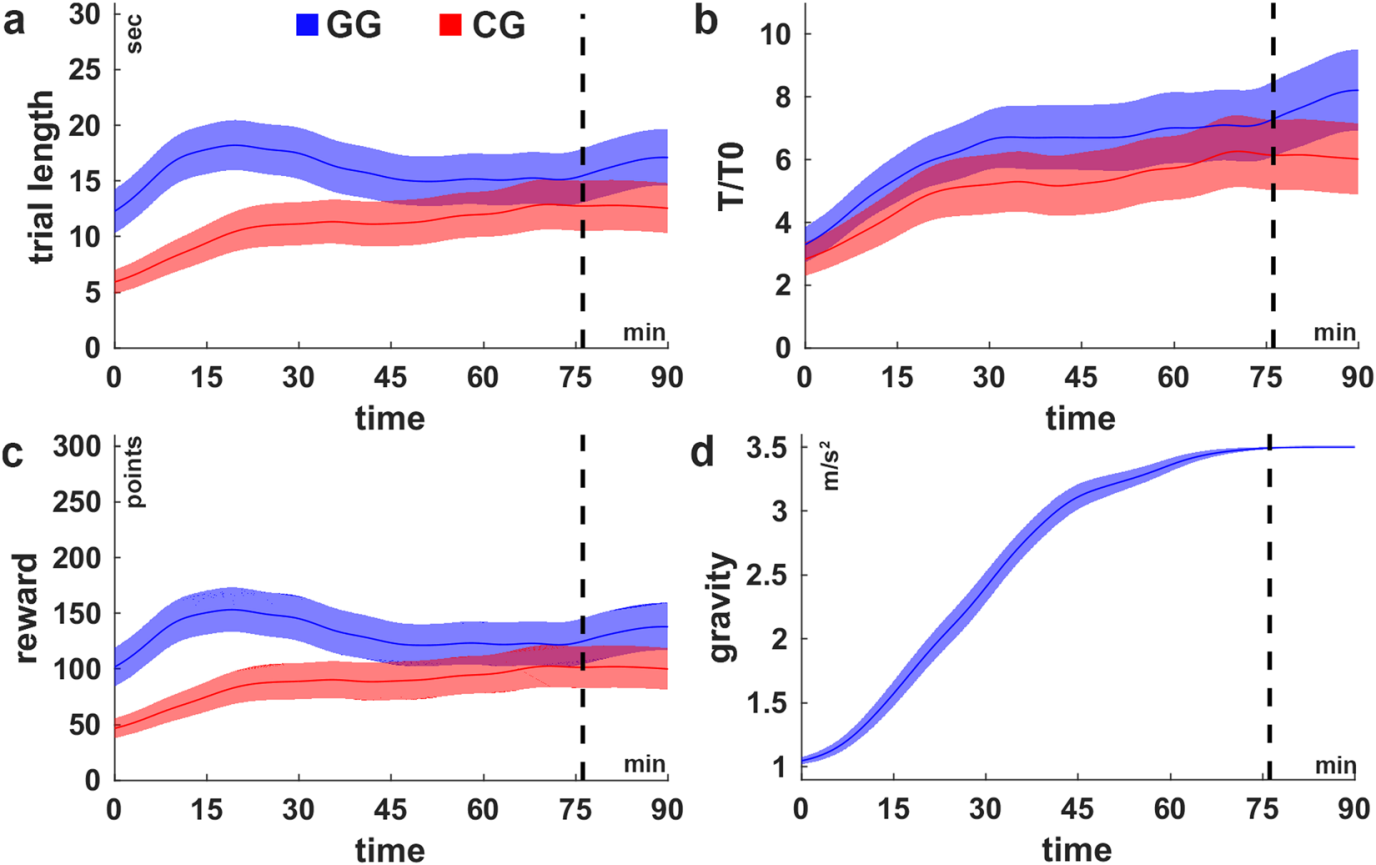
Learning curves. (**a**) Trial length, (**b**) normalized trial length (T/T_0_), (**c**) cumulated reward per trial across the experimental duration in the conditions GG (blue) and CG (red). The normalized trial length (**b**) reveals a monotonic improvement for both groups whereas the other measures (trial length and reward) are non-monotonic. (**d**) The average increase of the gravity of subject in condition GG. The shaded areas indicate the inter-subject-variability (±1SEM). The black dashed lines indicate the time at which all subjects in condition GG have reached maximum gravity (g_max_ = 3.5 m/s^2^) latest. For the purpose of illustration, the curves were smoothed over time using the weighted running average method.

Task performance, measured by the trial length, is shown in Fig. 2 as running average over the course of the experiment for both groups. Although the performance looks almost constant for the gradual group (Fig. 2a), keep in mind that the gravitational constant was increased after every successful trial making the task more difficult over time. In order to account for this influence, we also examined the improvement based on the normalized trial length T/T_0_ (Fig. 2b), which weights the measured trial length T using the theoretical trial length T_0_ that would be measured if no force were applied (see Methods). Thus, the normalized trial length expresses the task performance in comparison to doing nothing. Figure 2b shows that both groups improve monotonically according to this measure.

Comparing the average task performance within the first 5 minutes of the experiment reveals significantly higher performance for subjects in condition GG (trial length, Wilcoxon rank sum, p < 0.001). As the only difference between the groups is the difference in gravity during this phase, this result confirms the expectation that the gravity strongly influences the task difficulty. The difference in task performance between the groups persists however throughout the experiment. Finally, subjects in the gradual group are also significantly better at the end (last 5 minutes) of the experiment (trial length, Wilcoxon rank sum, p < 0.05), despite the fact that the gravity is identical for both groups at this point. The average course of the gravitational constant is shown in Fig. 2d.

We found no significant difference between the groups in T/T_0_ over the first 5 minutes (Wilcoxon rank sum, p = 0.15), which suggests that the difference found for the trial length is mostly because of the difference in gravity (difficulty) and is successfully cancelled out by the normalization. The normalization had no effect on the group difference at the end of the experiment, meaning that subjects in the gradual group are, also according to this measure, significantly better (Wilcoxon rank sum, p < 0.05). In summary, the gradual gravity condition facilitates the acquisition of the cart-pole balancing skill.

### Time of first success and frequency of early successes are related to end performance

An important incentive for improvement in acquiring a new skill is reward. The cumulative numeric reward at the end of the trial is shown in Fig. 2c. We found that this measure is mainly determined by the trial length, and does therefore not provide further insight.

Another form of reward is the balancing success (and failure). This is potentially an even stronger reward signal than the cumulative numeric reward because it represents the primary goal of the task.Subjects who manage to balance the system early during the experiment have more time to practice and reinforce this successful behaviour. We found that subjects in condition GG were significantly earlier able to balance the system for the first time (time of first successful trial, Wilcoxon rank sum, p < 0.001, median: GG = 2.8 min, CG = 24.2 min) and were thereby able to practice and explore the successful behaviour more deliberately in comparison to subjects in condition CG.

**Figure 3:**
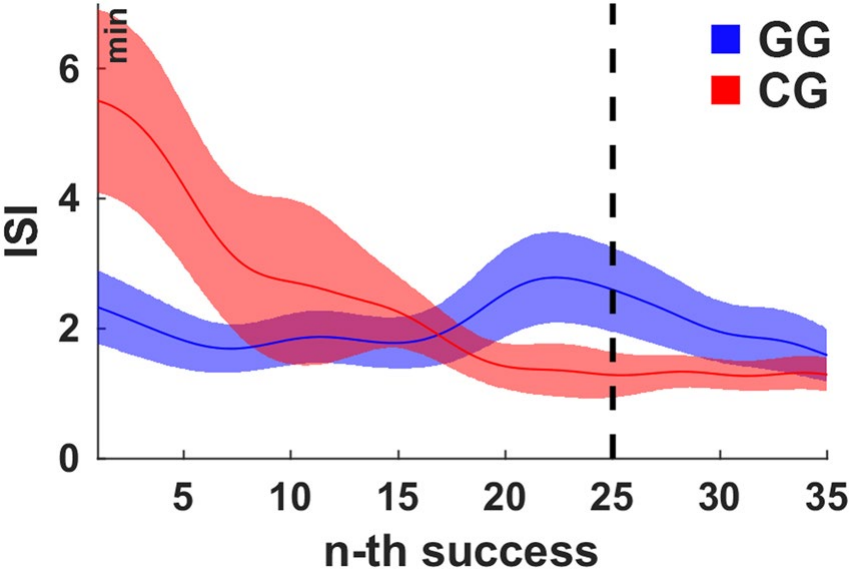
The average inter-success intervals for the first 35 successes. The inter-success intervals (ISI) in condition CG decrease monotonically whereas in condition GG an intermediate increase is observable just before subjects reached the maximum gravity after 25 successes. The shaded areas indicate the inter-subject-variability (±1 SEM). The black dashed line indicates the time at which all subjects in condition GG have reached maximum gravity (g_max_ = 3.5 m/s^2^) latest. For the purpose of illustration, the curves were smoothed over time using the weighted running average method.

The time between two successful trials (inter-success intervals) is yet another important factor for the reinforcement of behaviour because it expresses the frequency of success and thereby the frequency of reinforcement. Regression analysis using a linear mixed-effects model was conducted to examine the effect of group and index of success on the (log) inter-success interval (Fig. 3). Both factors as well as their interaction were significant (all p < 0.001). Post-hoc comparison of the groups however did not reveal any significant difference (p = 0.16), when the model was fitted over the first 35 success. To examine the very initial phase, we constrained the model to the first 20 successes. In addition to the significance of the factors and their interaction (all p < 0.001), the post-hoc comparison of the groups revealed now a significant difference (p < 0.01). Thus, in the beginning of the experiment, subjects in condition GG received more frequently positive reward (in form of success) than subjects in condition CG.

Correlation analysis within the groups revealed a significant relation (Spearman’s correlation coefficient, GG: rho = −0.57, p < 0.05; CG: rho = −0.59, p < 0.01) between the average inter-success internal over the first ten intervals and the task performance at the end of the experiment (average trial length over last 5 minutes). Performing the correlation analysis across both groups revealed the same relation (Spearman’s correlation coefficient, rho = −0.71, p < 0.001). Thus, subjects who are frequently successful at the beginning of the experiment show a high performance at the end, while subjects who are less frequently successful at the beginning show a low performance at the end. Overall, these results emphasize the importance of early and frequent success for achieving high performance towards the end.

### Subjects learn to perform actions more predictively and with less variability

**Figure 4:**
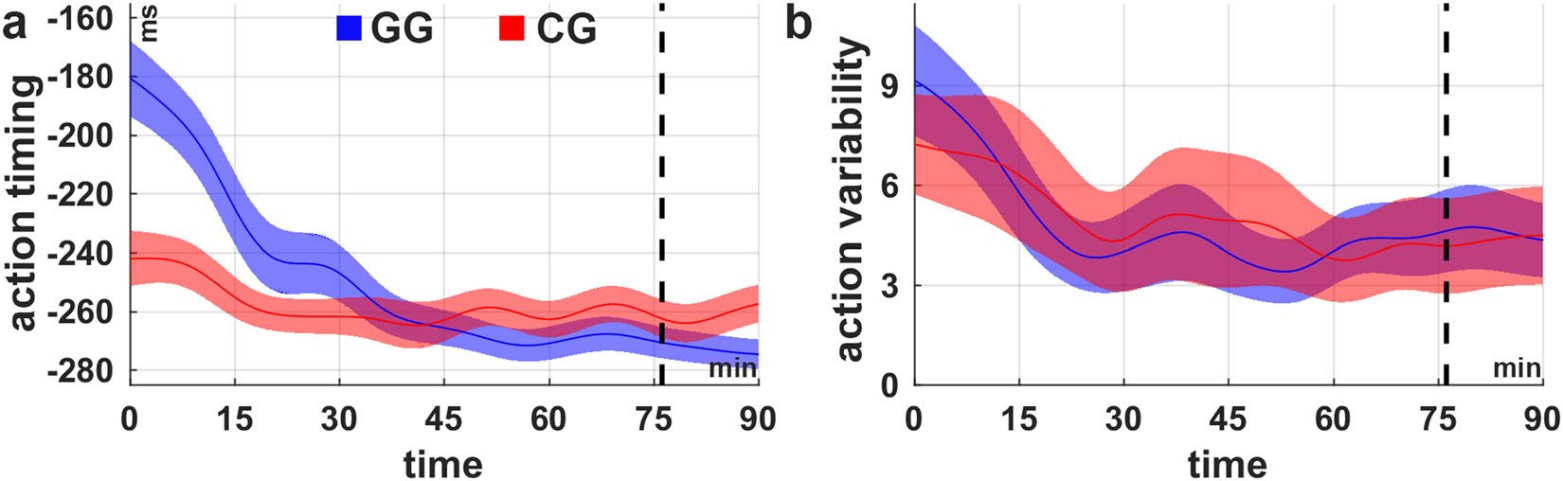
Action timing and variability over the course of learning. Average (**a**) action timing and (**b**) action variability over the course of the experiment for the conditions GG (blue) and CG (red). In both conditions the action timing as well as the action variability decline (actions are timed earlier, variability decreases) over time. Both measures were calculated in bins of 5 minutes length. The shaded areas indicate the inter-subject-variability (±1 SEM). The shaded areas indicate the inter-subject-variability (±1 SEM). The black dashed lines indicate the time at which all subjects in condition GG have reached maximum gravity (g_max_ = 3.5 m/s^2^) latest. For the purpose of illustration, the curves were smoothed over time using the weighted running average method.

We recorded the force, which the subjects applied to the cart as well as the state of the system for every frame. In order to analyse and quantify how subjects’ actions change over time, we defined two measures: the action timing and action variability. While the action timing describes the time when the force is changed relative to the occurrence of a certain pole angle (here, averaged over all pole angles), the action variability quantifies how consistently these actions are performed (see Methods for details). Analysis of the action timing over the course of the experiment (see Fig. 4a) revealed a monotonic change to earlier (more predictive) execution of actions (Spearman’s correlation coefficient, GG: rho = −0.55, p < 0.001; CG: rho = −0.15, p < 0.05). Similarly, correlation analysis revealed a significant decrease in action variability (Fig. 4b) over the course of the experiment in both conditions (Spearman’s correlation coefficient, GG: rho = −0.19, p < 0.002; CG: rho = −0.19, p < 0.002). Subjects learned to execute the actions earlier and thus more predictively with respect to the pole movement. Moreover, the performed actions are more consistent at the end of the experiment, which reflects subjects’ increased confidence about the high value of the specific action. These results strongly suggest that learning to perform actions predictively as well as decreasing the action variability are crucial components of acquiring the cart-pole balancing skill.

### Coherent modulation of the action timing, variability and task performance due to increments in gravity

**Figure 5:**
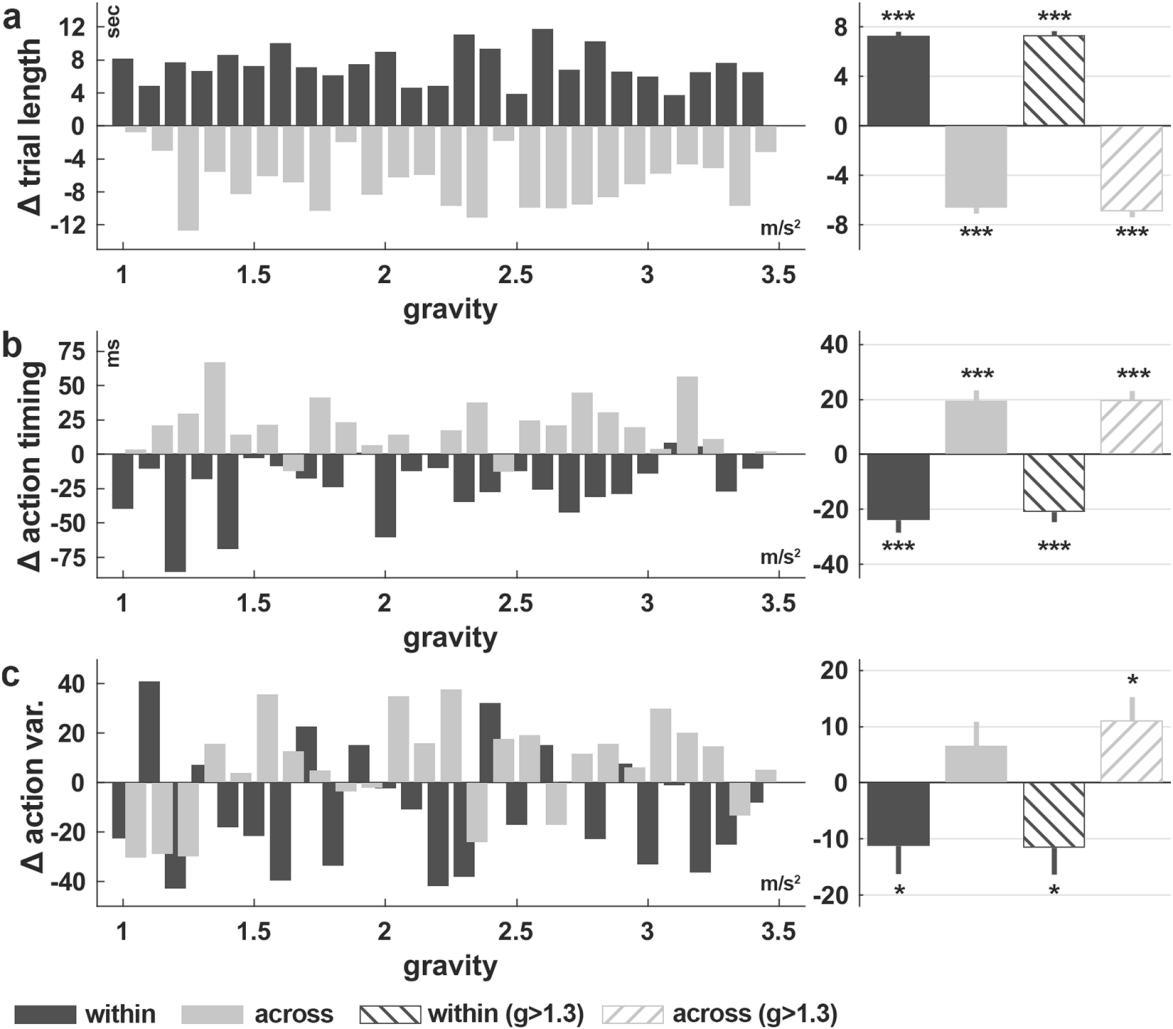
Change of trial length, action timing and variability as function of gravity changes (group GG). Change in (**a**) trial length, (**b**) action timing and (**c**) action variability for the subjects in the group GG. On the left, the measures are shown for each gravity step, and on the right, the average over all steps is shown for each measure with error bars (±1 SEM). Subjects improve (increase in trial length, more predictive actions, less variable actions) within and get worse (decrease in trial length, less predictive actions) across the gravity steps (see Methods and Fig. 7d). Excluding the first three gravity steps reveals also for the action variability a significant negative influence of the increments in the gravity. Significance codes: ***p < 0.001, *p < 0.05.

For subjects in condition GG, we analysed in detail how changes of the system dynamics influence the task performance, action timing and variability and as well as whether these are coherently modulated. Moreover, changes in the action timing would strongly indicate that subjects form a predictive model of the cart-pole system during the skill acquisition, which is adapted when the system dynamics change. Analysis of the average trial length (Fig. 5a) across and within gravity steps revealed significant improvement during phases of constant gravity (${\langle {P}_{2,g}-{P}_{1,g}\rangle }_{g}$, t-test, p < 0.001) and significant deterioration after an increment in gravity within the gravity steps (${\langle {P}_{1,g+1}-{P}_{2,g}\rangle }_{g}$, t-test, p < 0.001). Furthermore, we found that the action timing changes (Fig. 5b) towards an earlier (more predictive) execution of counteractive actions during phases of constant gravity (${\langle A{T}_{2,g}-A{T}_{1,g}\rangle }_{g}$, t-test, p < 0.001). Coherently with the changes in performance, the action timing is deteriorated after a change in gravity, i.e. actions are timed later, meaning rather in reaction to the events than predictive (${\langle A{T}_{1,g+1}-A{T}_{2,g}\rangle }_{g}$, t-test, p < 0.001). In alignment with these results, the action variability (Fig. 5c) decreases significantly during phases of constant gravity (t-test, p < 0.05) and increases after an increment in the gravity (for g > 1.3, t-test, p < 0.05). In summary, the task performance, action timing and variability are coherently modulated by the increments in gravity. This result strongly indicates that an internal model of the cart-pole dynamics is continuously re-calibrated and used for predictively controlling the system.

### Predictive action timing and low variability yield high task performance

**Figure 6:**
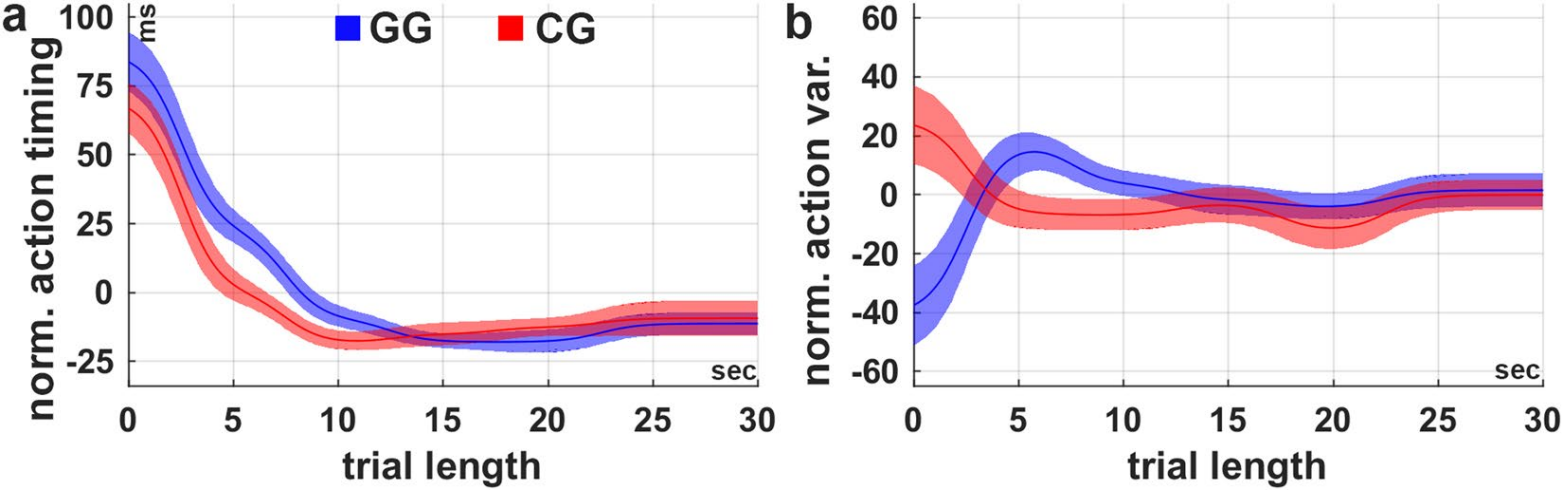
Relationship between task performance, normalized action timing and variability. Action timing and variability were normalized to remove the coherent influence of learning (see Methods: Relationship between action timing and task performance) and can therefore take negative values. (**a**) In both groups, there is a strong relationship between the normalized action timing and trial length (task performance). Generally, the more predictive actions are performed (negative action timing) the better the task performance. (**b**) Subjects in the group CG show a higher action variability for low task performances (trial length shorter than 5 seconds). The action variability is not significantly related to the task performance for subjects in the group GG. The shaded areas indicate the inter-subject variability (±1 SEM). For the purpose of illustration, the curves were smoothed over time using the weighted running average method.

The coherent modulation of the task performance, action timing and variability by changing the gravity indicates that predictive action timing is necessary for successfully balancing the pole. In order to investigate this aspect further and because all measures improve due to learning, we first subtracted the coherent influence of learning, yielding the normalized action timing and variability as function of the task performance (Fig. 6, see Methods for details). Correlation analysis revealed a significant relation between the trial length and normalized action timing (Fig. 6a) for both groups (Spearman’s correlation coefficient, GG: rho = −0.69, p < 0.001; CG: rho = −0.50, p < 0.001). Thus, the normalized action timing decreases significantly with increasing trial length, suggesting that actions need to be timed more predictively for high task performances than for low task performances. We found a similar correlation between the trial length and normalized action variability (Fig. 6b) in the condition CG. However, the relation seems to be much weaker and not present in the condition GG (Spearman’s correlation coefficient, GG: rho = 0.07, p = 0.50; CG: rho = −0.22, p < 0.05). Thus, in the group CG the task performance increases significantly with decreasing action variability, suggesting that the action variability needs to be low for high task performances. In summary, these results suggest that predictive timing of the actions and low variability is crucial for achieving high task performance.

## Discussion

The aim of this study was to elucidate how reward-based and error-based learning interact in one single task. We therefore investigated subjects’ learning behaviour in the cart-pole balancing task, which combines skill acquisition and internal model adaptation due to gradually changing dynamics of the cart-pole system. As hypothesized, we have found three main effects: (1) gradually increasing the difficulty of a task increases the amount and frequency of reward (success) early in the experiment, reinforcing the successful behaviour longer and benefiting skill acquisition. (2) When these changes influence the controlled system dynamics, there is not only a temporary decrease in general performance but also a specific degradation in the timing and variability of actions. Finally (3), performing actions predictively is crucial for successful cart-pole balancing.

### Gradual task-difficulty facilitates skill acquisition by means of success

Consistent with many studies on skill acquisition from a wide range of disciplines, we found that gradually increasing the difficulty of a task is beneficial for acquiring a motor skill. Choosing an adequate initial task complexity and increasing the complexity in relation to the improvement during learning is seen as important prerequisite for efficient motor learning and is, thus, commonly used as training principle in sports as well as in rehabilitation^16,45^.

The theoretical relationship between task difficulty, skill level and learning was systematically described in the “challenge point” framework^46^. The optimal challenge point characterizes the optimal task difficulty for learning at a certain skill level. Guadagnoli and Lee^46^ distinguished between the (a) nominal and (b) functional task difficulty. While the nominal task difficulty is a feature of the task, the functional task difficulty depends in addition to the task also on the skill level of the learner. As part of their framework, they relate the task difficulty and skill to the amount and ability to process information. They elaborate that learning is inefficient if the amount of information is too high (i.e. functional task difficulty is too high), or the capability to process the available information is too low (i.e. skill level is too low). Thus, regarding the efficiency of learning, there is an optimal rate of increasing the task difficulty, which depends on the learner’s skill level.

Consistent with this framework, novices in our experiment benefited from an initially low gravity level, yielding low time-constraints for the control of the pole (see Figure S2). By increasing the gravity after every success, we increased the task difficulty in relation to the skill level and maintained the challenge of the task. In particular, by tightening the time-constraints we gradually encouraged the use of predictive control, which was also identified as crucial mechanism for successfully controlling the cart-pole system.

Despite these more theoretical frameworks and observational studies, mainly in sport science, there is a limited number of studies investigating the effects of adaptively increasing the task complexity on motor learning processes. Most related is a study by Choi *et al*.^38^ in which the authors examined retention of different visuomotor transformations over 4 days. They found that the retention is largely improved by adapting the number of trials as a function of performance in comparison to maintaining fixed random scheduling. Varying the difficulty, in terms of limiting the maximum movement time, as a function of performance also improved learning, although to a lesser extent. Since further studies on this topic are lacking, an interesting and still open research question is how the task difficulty has to be increased in order to maintain continuous optimal challenge. Especially for tasks like ours, in which the increase of task difficulty also results in a change of object dynamics, this is an intriguing question. In this case, optimal training schedules have also to take into account that adaptation to changes in object dynamics are necessary. Fewer increments in gravity imply fewer adaptations but at the cost of large increments in task complexity, which might influence reward and motivation negatively.

### Internal forward models support the predictive control of object dynamics

In addition to the substantial number of studies examining internal model adaptation in classical visuomotor adaptation paradigms, several studies have also shown that humans learn and maintain internal models of object dynamics for optimizing their actions and the corresponding sensory consequences^3,29,31,47–50^. For balancing an inverted pendulum, for example, Mehta and Schaal^31^ have demonstrated that actions of trained subjects performed during short absence of visual feedback (450–550 ms) do not significantly differ from actions with visual feedback. They suggested that predictions formed by an internal forward model of the cart-pole system replace the visual feedback during these phases. Our results are in line with this hypothesis and imply predictive control as a critical mechanism for successfully mastering the cart pole task. In our experiment, subjects performed the actions progressively in advance in relation to the state of the system (Fig. 4a), which indicates gradually improved predictive behaviour. Emphasizing the importance of action timing even further, we demonstrated a crucial relationship between the measured action timing and cart-pole balancing performance (Fig. 6a).

### The adaptation of forward models during the gradual increase of gravity

In order to profit most from internal forward models of object dynamics, they have to be acquired in the beginning of the learning process and need to be adapted when the behaviour of the modelled object changes^32^. Intriguingly, when interacting with novel objects, people seem to learn to predict the behaviour of the objects before they can master its control^47^. For real balancing of a pole on the fingertip, Lee and colleagues^44^ suggested that subjects acquire an accurate pendulum model, which accounts for the gravitational dynamics and mass distribution along the pole, already within the first moments of wielding the pole through “dynamic touch”^51^.

On the other hand, changes to the object dynamics, such as increments in gravity in our experiment, result in inaccurate extrapolations of the system’s state, which primarily leads to deteriorated performance (Fig. 5a). A more specific prediction is that the acceleration of the pole is underestimated after an increase of the gravity. Thus, with respect to a certain pole angle, responses would be delayed. Our analysis of the change in action timing across the gravity steps (${\langle A{T}_{1,g+1}-A{T}_{2,g}\rangle }_{g}$) supports this hypothesis (Fig. 5b). Consequently, the altered system dynamics and the now imprecise predictions of the forward model lead to increased spatio-temporal variability (Fig. 5c). These observations are consistent with previous studies showing temporarily increased spatio-temporal variability in virtual bouncing game after changing gravity^52^. As soon as the internal model is adapted to the new dynamics of the object, state estimates are again accurate, leading to normal motor variability and performance.

### Gradual increase of difficulty in skill acquisition tasks and sensory-motor adaptation paradigms

In recent years, increasing attention has been gained by sensorimotor adaptation studies which examine the effects of perturbing the sensorimotor mapping of reaching gradually instead of suddenly^8,9,53,54^. The (performance-independent) gradual perturbation schedule does not predominantly facilitate learning, but leads to an increased and prolonged after-effect after removing the perturbation^8^. It has been suggested that gradual perturbation schedules favour implicit learning mechanisms (involving internal forward models) in contrast to explicit rule-based learning^53^.

Even more influential are gradual perturbation paradigms for sensorimotor adaptation when binary reward-based feedback is provided instead of end-point errors in reaching. In such paradigms only hitting the target is positively rewarded, which is difficult if perturbations are large and sudden^10–12^. Thus, finding the right correction for the perturbation and receiving positive reward is difficult, which makes reward-based learning tough. In contrast, if perturbations are gradual, the necessary adaptation between subsequent perturbation steps requires less exploration and may even be in the order of magnitude as subjects’ motor variability^55,56^, leading to implicit learning and fast adaptation of the underlying internal forward models. From a computational point of view, instead of searching for an adequate solution, which is in the sensory-action space faraway, the gradual perturbation paradigm allows for guided exploration in small steps with intermittent reward.

This view is transferable to our task when task complexity is gradually increased. Based on simple heuristic control rules (explicit knowledge), which are sufficient to solve the problem (balance the pole) under the easy initial task condition of low gravity (g_0_ = 1.0 m/s^2^), the exploration during learning is guided stepwise through the sensory-action space to a solution for the complex task condition (g_max_ = 3.5 m/s^2^). The potential benefits of this approach are supported from previous results from machine learning, showing that the use of a fuzzy controller to implement heuristic knowledge and reward-based learning mechanisms for the refinement of control strategies can facilitate the learning processes substantially^57^. By initializing and guiding the exploration of the sensory-action space, subjects receive primarily more frequently positive reward, especially early in the learning process.

Success and reward have shown to be strong reinforcement signals, which can not only accelerate learning^12,58^ but also have a motivational influence on the subject^59,60^. Moreover, it has been shown that offline gains and long-term retention of newly formed motor memories benefit from training under rewarding conditions^36^, demonstrating the power of reward. Recently, it was shown by Therrien *et al*.^11^, that adaptation to sudden sensorimotor perturbations is possible with binary reward, if the reward is provided relative to the current mean performance (closed loop reinforcement). Hence, instead of providing positive reward only if the target was hit, reward is used to guide the exploration towards the target, by providing positive reward if the action was better than the previous ones. Thus, instead of changing the task demands like in our approach, the reward landscape is shaped by the current performance. Both approaches demonstrate the benefit of incorporating the functional task difficulty^46^, which is set by the current skill level of the learner, into the design of training schedules.

### Motor skill acquisition as an interaction of multiple learning mechanisms

In this study, we focused our analysis on the interaction of reward- and error-based learning mechanisms when the gravity is changed during the acquisition of the cart-pole balancing skill. In addition to this specific interaction of these two learning mechanisms, there may exist several further interactions involving other learning mechanisms and different kinds (e.g. implicit or explicit) of knowledge representations.

For example, based on other tasks or by exploiting the basic understanding of physics, explicit control knowledge could be formed. By exploiting this knowledge for the initialization of actions, the exploration and thereby reward-based learning can be biased considerably. Depending on the validity of the derived control knowledge, learning can be facilitated or slowed down.

Furthermore, use-dependent learning may contribute in addition to reward- and error-based learning and may interact with these two learning mechanisms^61,62^, especially by biasing the choice of explored actions^63^. However, a reliable distinction between use-dependent and reward-based learning is hard to find in the balancing task because of the permanent presence of the reward signals.

## Conclusions and Outlook

In this study, we have presented an experimental setup to investigate reward-based motor skill acquisition to control an external object with changing dynamics.

We conclude that gradual increase in task difficulty accompanied with changes in object dynamics facilitates, despite interleaved brief degradations in performance, the skill acquisition by means of success-mediated learning. Interleaved degradation in performances due to changes in object dynamics is associated with less predictive timing and increased variability of actions. The presented results motivate several further studies, in order to examine the interplay between reward-based skill acquisition and adaptation of the internal model to changes of the object dynamics in more detail. As interesting open questions remain for instance (i) potential after-effects in timing after the gravity is changed back to a lower value, (ii) differences in retention between gradual and sudden increase of gravity as well as (iii) finding an optimal schedule to increase the gravity, in order to minimize the number of changes while preserving the advantage of slowly increasing the complexity. Future studies will have to examine the neural mechanisms of skill acquisition when task difficulty and object dynamics are gradually changed. In particular, the cerebellum, with its role in reward-based learning^64^ and maintenance of internal models of object dynamics, might be an interesting target of further investigation. In conclusion, these studies will advance our knowledge in skill acquisition for complex movements, applicable in several disciplines, such as sports, professional skill development, and neuro rehabilitation.

## Methods

### Subjects

We analysed thirty right-handed subjects (age range 18–30 years, mean age 23.4; 15 females, 15 males).

All subjects gave informed written consent prior to participation. The study was carried out according to standard guidelines and regulations and had been approved by the ethical review board of the medical faculty of the Eberhard-Karls-University and university clinics in Tübingen, Germany (AZ 409/2014BO2). Subjects were randomly assigned to one of two groups corresponding to the two examined experimental conditions (gradual gravity, GG; constant gravity, CG; see Learning Paradigm). Both groups consisted of 15 subjects with similar average age (GG: 24.1 years, CG: 22.6 years). Gender was balanced between groups (GG: 9 males, 6 females, CG: 7 males, 8 females). All subjects were right-handed and used their right hand throughout the experiment. Subjects were reimbursed independent of performance.

### Details of Experimental Setup

The virtual cart-pole system consists of a cart to which a one-meter long pole is attached. Due to the assigned masses (pole: 0.08 kg, cart: 0.4 kg) gravity forces the pole to rotate downwards when not being perfectly upright. We did not simulate friction. Forces from the left or right are applied by the subjects to the cart in order to control the system. Participants controlled the virtual force using a SpaceMouse® Pro (3Dconnexion, Fig. 1b). This input device is similar to a joystick but it can measure six degrees of freedom (DOF) including the left-right translation. The device’s knob can be displaced ±1.5mm in this direction and exerts a force of 7.4 N at full lateral displacement back to the rest position in the centre. We used this DOF to control the virtual force. To this end, the lateral displacement of the device’s knob relative to the rest position in the centre of the device was translated into a virtual force into the same direction with proportional magnitude (see below) that pushes against the cart. Hence, a rightward knob movement causes a virtual force, which pushes the cart to the right. The simulation of the cart-pole dynamics was implemented in MATLAB (The MathWorks, Inc.) using the 4^th^-order Runge-Kutta method. Visual feedback was provided on a 15 inch monitor using the Psychtoolbox^65–67^ at a refresh rate of 60 Hz. Correspondingly, the time-discretization constant Δt of the simulation was set to 1/60 s. Subjects were not constrained in posture but were asked to sit comfortable about 60 cm away from the monitor. The input-device was aligned with the monitor such that the left-right knob movement was in correspondence with the virtual force and cart movement on the monitor.

### Learning Paradigm and Experimental Conditions

At the beginning of every trial the cart-pole system is initialized with a random pole angle drawn uniformly from [−7.5, 7.5] degrees, positioned at the centre of the track with both velocities (cart and angular pole velocity) set to zero.

The pole has to remain within the green circular segment (±60 degree, Fig. 1a) while the cart must not leave the track (±5 m). This has to be achieved by applying forces of up to 4 N from either side to the cart using the input device. The forces accelerate the cart, depending on the direction, to the left or right by which the pole can be balanced. A trial was considered successful, if balance was maintained for 30 seconds without violating any constraint. Hence, trials were at maximum 30 seconds long. Violation of one of the constraints (cart position, pole angle) also terminates the trial. The number of trials was not limited, instead we limited the duration of the experiment (see below). Before the next trial begins, feedback about the violated constraint and the duration of the trial is provided.

In addition to the terminal feedback at the end of every trial, subjects also receive cumulative reward during the trial, which is displayed as number in the cart and updated in every frame (Fig. 1a). The theoretically maximum reward per second is 10 points, which can be achieved by holding the pole perfectly vertical, keeping the cart exactly in the centre of the track while not applying any force to the cart (for details, see S1 Appendix). Thus, in theory, a maximum reward of 300 points per trial is achievable. This ultimately means that subject has to keep the system within the constraints for 30 seconds, i.e. balance the pole on the cart for 30 seconds without leaving the track. Subjects were instructed about these factors and were asked to maximize the reward in every single trial.

### Data Processing and Analysis

We analysed the trial length, reward per second and cumulative reward of every trial (including the unsuccessful balancing attempts) in order to quantify improvement. The trial length T can be characterized by two factors: (i) T_0_, which is the time it takes for the pole to violate the pole angle constraint when no controlling input would be present (Figure S1) and, (ii) the time by which the trial is prolonged due to subject’s actions. The time T_0_ is completely described by the initial pole angle and the gravity. In a low-gravity environment (e.g. at the beginning of condition GG) the time T_0_ is much longer than for higher gravity values (e.g. throughout condition CG or at the end in condition GG). Hence, in condition GG T_0_ decreases as function of the increasing gravity during the experiment and progressively limits the time in which subjects have to counteract (tightening of time-constraint). We simulated the system with different initial pole angles and thereby revealed a nonlinear relation between T_0_ and the gravity (Figure S1). In order to account for this factor, we determined T_0_ for every recorded trial and calculated the ratio T/T_0_, which is a measure of how much better (>1) or worse (<1) the subject was controlling the system compared to doing nothing.

Other important factors, especially in the context of reward-based learning, are reward, success and the time passed between subsequent successes. In the following, we call the latter inter-success intervals (ISI).

### Determining the action timing and variability

**Figure 7:**
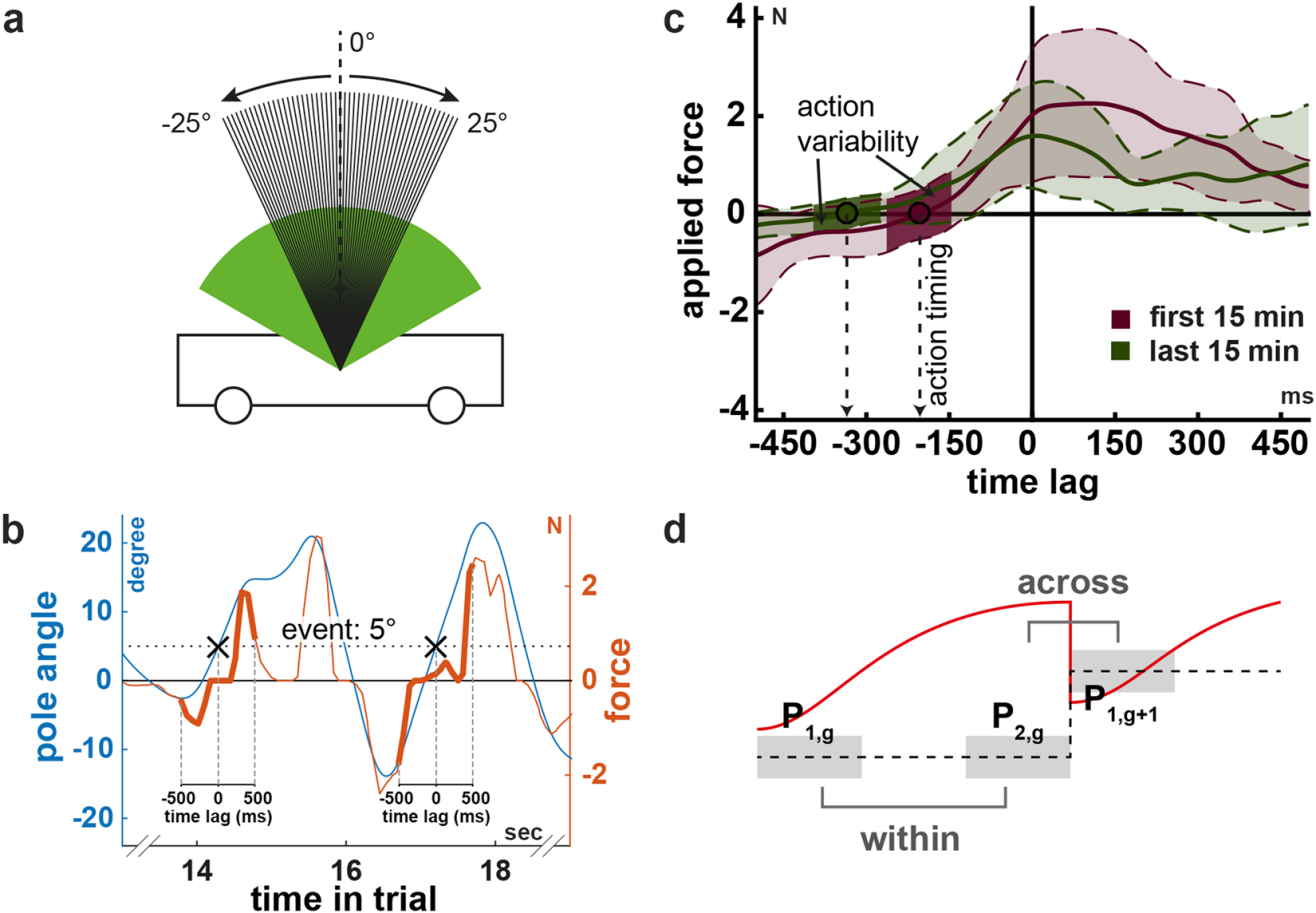
Schematic of the action timing and the change of measures as function of the gravity. (**a**) All pole angles investigated as events (integer valued pole angles from −25 to 25°). The arrows indicate the direction of the pole movement. (**b**) Pole angle (blue) and input force (orange) trajectories in a representative trial illustrating two event occurrences (black crosses) and corresponding two force segments (thick lines). Negative force values correspond to a leftwards force. Aligning all segments that correspond to one event and averaging the segments over different trials in a window of 2 minutes yields similar curves to those shown in panel c. (**c**) Average force segments of a representative subject for two periods during learning (first 15 minutes: purple, last 15 minutes: dark green) for illustrating the action timing and variability measures. The circles indicate the time (action timing) when the subjects changed the direction of the force relative to the occurrence of the event (zero time lag). Negative and positive time lags represent the time before and after the event. As expected, early (first 15 minutes, purple) during learning actions are performed rather in reaction to event occurrences (towards positive time lags) whereas learning leads to the ability to make actions predictively (more negative time lag, dark green). Coloured areas illustrates the variability in force segments. The dark coloured areas indicate the action variability. It is the average standard deviation of the force segments ± 60ms around the zero crossing (action timing). The variance in input force round the zero crossing (action variability) is lower for actions late during learning (last 15 minutes), suggesting more consistency. (**d**) Illustration of the procedure to examine changes in different measures relative to an increase in gravity. We here show exemplarily the trial length (red curve) in relation to the gravity (dashed line). The light grey areas illustrate the periods under investigation. Subtracting the average trial length in the highlighted periods yields the change within (P_2,g_ − P_1,g_) and across (P_1,g+1_ − P_2,g_) the gravity step(s).

In order to infer more about the underlying mechanisms of improvement, we quantified the changes in subjects’ control policies. We therefore examined the applied forces as function of the system state, specifically as function of the pole angle (Fig. 7a). The rationale of our approach is to define events in the state space and analyse the applied forces relative to those events (event-triggered averaging, Fig. 7b,c). We focus our analysis on the situations when the pole is tilted by a certain angle and is rotating downwards. In these situations, which we describe as events, a counter-action is necessary. Using our method we determined when and how variable these counter-actions were performed. The following four steps describe the procedure in more detail.

i. We defined the integer valued pole angles in the range from −25 to 25 degrees as events (Fig. 7a). For each of these 51 events we determined the occurrences in every trial. Event occurrences between two time-discretization steps (frames) were estimated using linear interpolation.
ii. We then excluded all event occurrences in which the pole is actually rotating upwards and therefore no counter-action is required. We further excluded extreme pole angle velocities. To this end, we excluded all event occurrences in a running window of two minutes across trials, for which the pole angle velocity did not lay between the 20%- and 80%-quantiles of all observed pole angle velocities.
iii. Next, we extracted segments of one-second length from the force input trajectory, which are centred on the previously determined event occurrences, meaning that half of the segment happed before (negative time-lag) and the other half happened after (positive time-lag) the event occurred (Fig. 7b). These segments look roughly like sigmoidal functions going from negative to positive force values or vice versa, corresponding to a left-right or right-left movement of the device knob by the subject.
iv. In the last step, we averaged all segments corresponding to one event (pole angle) in a running window of 2 minutes length across trials and determined the zero crossing of the average segment (Fig. 7c). Thereby we find the time of change from a negative (leftwards) to a positive (rightwards) force (or vice versa) relative to the event occurrence (zero time lag).

We refer to this time (when the actions change relative to the state of the system) as action timing. Notice, that we do not interpret this measure as the reaction time, even though it might be related. Furthermore, we estimated the variability of the actions by calculating the mean standard deviation of the applied forces in a centred window of 120ms length around the zero crossing (Fig. 7c).

### Relationship between action timing and task performance

During the analysis of the influence of the action timing and variability on the task performance, we faced the problem that all three measures change over time due to learning. Correlation analysis between these measures therefore trivially revealed high correlation. We were however interested in verifying the action timing and variability as crucial measures for achieving high balancing performance. Hence, we were looking for a normalization, which eliminates the concurrent influence of learning on all measures but preserves potentially existing correlations between the measures. Since we did not measure learning per se, we had to use time as representative. In order to express the action timing and action variability as functions of task performance (trial length) and time, we discretized trial length and time (Figure S2a). Trial length was split into bins of 5 seconds, while time was split into bins of 5 minutes. Within each of the two-dimensional bins, the action timing and action variability have been determined. We eliminated the influence of learning by subtracting the average action timing (action variability respectively) across all trial length bins within each time bin (Figure S2a and b). The action timing and variability are thereby expressed as function of the discretized trial length, independent of the influence of learning. We call the resulting measures *normalized* action timing and *normalized* action variability. Due to the subtraction of the average, the normalized action variability can take negative values.

### Quantification of changes induced by gradual increments in gravity

We next analysed the influence of changing the gravitational constant on the performance, action timing and motor variability in the gradual group. The gravitational constant is described by a monotonically increasing stairs function over time (Fig. 1c). Consequently, there are phases of constant value and sudden but small changes from one trial to the next (Fig. 7d). Let P_1,g_ denote the average trial length during the first third of the gravity step with value *g* and let P_2,g_ denote the average trial length during the last third of this step. We estimated the improvement within gravity steps by taking the difference P_2,g_ − P_1,g_. Averaging these across all steps yields a single value ${\langle {P}_{2,g}-{P}_{1,g}\rangle }_{g}$ for each subject, representing the average improvement during periods in which the system does not change. Similarly, we estimated the influence of an increment in gravity by calculating the average trial length over the last third (P_2,g_) and the first third of the next step (P_1,g+1_). The average difference over all gravity steps ${\langle {P}_{1,g+1}-{P}_{2,g}\rangle }_{g}$ measures the average change in performance caused by the changes of the system dynamics. Notice, that we denote the average of a measure across all gravity steps *g* by ${\langle \cdot \rangle }_{g}$.

For the action timing and variability, we proceeded similarly but calculated the differences for each event (pole angle) separately and then averaged over all events and gravity steps. Thereby we can analyse changes in the action timing (AT) and variability (AV) during periods of constant system dynamics (${\langle A{T}_{2,g}-A{T}_{1,g}\rangle }_{g}$ and ${\langle A{V}_{2,g}-A{V}_{1,g}\rangle }_{g}$) and the influence of sudden changes (${\langle A{T}_{1,g+1}-{P}_{2,g}\rangle }_{g}$ and ${\langle A{V}_{1,g+1}-A{V}_{2,g}\rangle }_{g}$).

### Statistics

All statistical analyses were performed in R (v3.3.2) using the package lme4 (v1.1), lmerTest (v2.0), phia (v0.2). Significance tests for differences between populations were performed using the two-sided t-test if measures were normally distributed according to the Shapiro-Wilk-Test. Otherwise Wilcoxon’s rank-sum test for equal medians has been used. Relationship between measures was evaluated using Pearson’s linear correlation coefficient if measures were normally distributed according to the Shapiro-Wilk-Test. Otherwise Spearman’s correlation coefficient was used. For the analysis of the measures over time, correlation coefficients were computed or linear mixed-effects models were used, which included a random effect for the intercept of each subject.

### Data Availability

All data generated or analysed during this study are included in this published article (and its Supplementary Information files).

## Electronic supplementary material

Supplementary Material 1

Supplementary Material 2

Dataset 1

Dataset 2

Dataset 3

Dataset 4

Dataset 5

Dataset 6

## References

[1] Prablanc C, Tzavaras A, Jeannerod M (1975). Adaptation of hand tracking to rotated visual coordinates. Perception & Psychophysics.

[2] Shadmehr R, Smith MA, Krakauer JW (2010). Error Correction, Sensory Prediction, and Adaptation in Motor Control. Annual Review of Neuroscience.

[3] Shadmehr R, Mussa-Ivaldi FA (1994). Adaptive representation of dynamics during learning of a motor task. The Journal of Neuroscience.

[4] Bastian AJ (2006). Learning to predict the future: the cerebellum adapts feedforward movement control. Current Opinion in Neurobiology.

[5] Blakemore S-J, Wolpert DM, Frith C (2000). Why can’t you tickle yourself?. Neuroreport.

[6] Blakemore S-J, Frith CD, Wolpert DM (2001). The cerebellum is involved in predicting the sensory consequences of action. Neuroreport.

[7] Tseng Y, Diedrichsen J, Krakauer JW, Shadmehr R, Bastian AJ (2007). Sensory Prediction Errors Drive Cerebellum-Dependent Adaptation of Reaching. Journal of Neurophysiology.

[8] Kagerer FA, Contreras-Vidal JL, Stelmach GE (1997). Adaptation to gradual as compared with sudden visuo-motor distortions. Experimental Brain Research.

[9] Orban de Xivry J-J, Lefèvre P (2015). Formation of model-free motor memories during motor adaptation depends on perturbation schedule. Journal of Neurophysiology.

[10] Izawa J, Shadmehr R (2011). Learning from Sensory and Reward Prediction Errors during Motor Adaptation. PLoS Computational Biology.

[11] Therrien AS, Wolpert DM, Bastian AJ (2016). Effective reinforcement learning following cerebellar damage requires a balance between exploration and motor noise. Brain.

[12] Nikooyan AA, Ahmed AA (2015). Reward feedback accelerates motor learning. Journal of Neurophysiology.

[13] Sternad D, Huber ME, Kuznetsov N (2014). Acquisition of novel and complex motor skills: stable solutions where intrinsic noise matters less. Advances in Experimental Medicine and Biology.

[14] Newell KM (1991). Motor skill acquisition. Annual Review of Psychology.

[15] Shmuelof L, Krakauer JW, Mazzoni P (2012). How is a motor skill learned? Change and invariance at the levels of task success and trajectory control. Journal of Neurophysiology.

[16] Schmidt, R.A. & Lee, T.D. *Motor control and learning. A behavioral emphasis*. 5th ed. (Human Kinetics, Champaign, IL, 2011).

[17] Yarrow K, Brown P, Krakauer JW (2009). Inside the brain of an elite athlete: the neural processes that support high achievement in sports. Nature Reviews Neuroscience.

[18] Reis J (2009). Noninvasive cortical stimulation enhances motor skill acquisition over multiple days through an effect on consolidation. Proceedings of the National Academy of Sciences.

[19] Hasson CJ, Shen T, Sternad D (2012). Energy margins in dynamic object manipulation. Journal of Neurophysiology.

[20] Ronsse R, Sternad D (2010). Bouncing between model and data: stability, passivity, and optimality in hybrid dynamics. Journal of Motor Behavior.

[21] Sternad D, Duarte M, Katsumata H, Schaal S (2001). Dynamics of a bouncing ball in human performance. Physical review. E, Statistical, nonlinear, and soft matter physics.

[22] Abe MO, Sternad D (2013). Directionality in distribution and temporal structure of variability in skill acquisition. Frontiers in Human Neuroscience.

[23] Davidson PR, Wolpert DM (2003). Motor learning and prediction in a variable environment. Current Opinion in Neurobiology.

[24] Desmurget M, Grafton S (2000). Forward modeling allows feedback control for fast reaching movements. Trends in Cognitive Sciences.

[25] Miall RC, Christensen LOD, Cain O, Stanley J (2007). Disruption of State Estimation in the Human Lateral Cerebellum. PLoS Biology.

[26] Wolpert DM, Ghahramani Z, Flanagan JR (2001). Perspectives and problems in motor learning. Trends in Cognitive Sciences.

[27] Kurtzer I, Pruszynski JA, Scott SH (2008). Long-Latency Reflexes of the Human Arm Reflect an Internal Model of Limb Dynamics. Current Biology.

[28] Dingwell JB, Mah CD, Mussa-Ivaldi FA (2002). Manipulating Objects With Internal Degrees of Freedom: Evidence for Model-Based Control. Journal of Neurophysiology.

[29] Mah CD, Mussa-Ivaldi FA (2003). Evidence for a specific internal representation of motion-force relationships during object manipulation. Biological Cybernetics.

[30] Ahmed AA, Wolpert DM, Flanagan JR (2008). Flexible representations of dynamics are used in object manipulation. Current Biology.

[31] Mehta B, Schaal S (2002). Forward Models in Visuomotor Control. Journal of Neurophysiology.

[32] Wolpert DM, Flanagan JR (2001). Motor prediction. Current Biology.

[33] Buszard T, Farrow D, Reid M, Masters RSW (2014). Modifying equipment in early skill development: a tennis perspective. Research Quarterly for Exercise and Sport.

[34] Buszard T, Reid M, Masters R, Farrow D (2016). Scaling the Equipment and Play Area in Children’s Sport to improve Motor Skill Acquisition: A Systematic Review. Sports medicine.

[35] Farrow D, Reid M (2010). The effect of equipment scaling on the skill acquisition of beginning tennis players. Journal of Sports Sciences.

[36] Abe M (2011). Reward improves long-term retention of a motor memory through induction of offline memory gains. Current Biology.

[37] Badami R, VaezMousavi M, Wulf G, Namazizadeh M (2011). Feedback after good versus poor trials affects intrinsic motivation. Research Quarterly for Exercise and Sport.

[38] Choi Y, Qi F, Gordon J, Schweighofer N (2008). Performance-Based Adaptive Schedules Enhance Motor Learning. Journal of Motor Behavior.

[39] Barto AG, Sutton RS, Anderson CW (1983). Neuronlike adaptive elements that can solve difficult learning control problems. IEEE Transactions on Systems, Man, and Cybernetics.

[40] Milton J (2009). The time-delayed inverted pendulum: Implications for human balance control. Chaos.

[41] Balasubramaniam, R. In *Progress in Motor Control*, edited by M. J. Richardson, M. A. Riley & K. Shockley 149–168 (Springer New York, 2013).

[42] Cluff T, Boulet J, Balasubramaniam R (2011). Learning a stick-balancing task involves task-specific coupling between posture and hand displacements. Experimental Brain Research.

[43] Foo P, Kelso JAS, Guzman GC (2000). Functional stabilization of unstable fixed points: Human pole balancing using time-to-balance information. Journal of Experimental Psychology: Human Perception and Performance.

[44] Lee K-Y, O’Dwyer N, Halaki M, Smith R (2015). Perceptual and motor learning underlies human stick-balancing skill. Journal of Neurophysiology.

[45] Nielsen JB, Willerslev-Olsen M, Christiansen L, Lundbye-Jensen J, Lorentzen J (2015). Science-based neurorehabilitation: recommendations for neurorehabilitation from basic science. Journal of Motor Behavior.

[46] Guadagnoli MA, Lee TD (2004). Challenge point: a framework for conceptualizing the effects of various practice conditions in motor learning. Journal of Motor Behavior.

[47] Flanagan JR, Vetter P, Johansson RS, Wolpert DM (2003). Prediction Precedes Control in Motor Learning. Current Biology.

[48] Flanagan JR, Wing AM (1997). The Role of Internal Models in Motion Planning and Control: Evidence from Grip Force Adjustments during Movements of Hand-Held Loads. The Journal of Neuroscience.

[49] Kawato M (1999). Internal models for motor control and trajectory planning. Current Opinion in Neurobiology.

[50] Dingwell JB, Mah CD, Mussa-Ivaldi FA (2003). Experimentally Confirmed Mathematical Model for Human Control of a Non-Rigid Object. Journal of Neurophysiology.

[51] Turvey MT (1996). Dynamic touch. American Psychologist.

[52] Ronsse R, Wei K, Sternad D (2010). Optimal control of a hybrid rhythmic-discrete task: the bouncing ball revisited. Journal of Neurophysiology.

[53] Taylor JA, Krakauer JW, Ivry RB (2014). Explicit and implicit contributions to learning in a sensorimotor adaptation task. The Journal of Neuroscience.

[54] Criscimagna-Hemminger SE, Bastian AJ, Shadmehr R (2010). Size of Error Affects Cerebellar Contributions to Motor Learning. Journal of Neurophysiology.

[55] Torres-Oviedo G, Bastian AJ (2012). Natural error patterns enable transfer of motor learning to novel contexts. Journal of Neurophysiology.

[56] Wu HG, Miyamoto YR, Castro LuisNicolasGonzalez, Ölveczky BP, Smith MA (2014). Temporal structure of motor variability is dynamically regulated and predicts motor learning ability. Nature Neuroscience.

[57] Berenji HR, Khedkar P (1992). Learning and tuning fuzzy logic controllers through reinforcements. IEEE Transactions on Neural Networks.

[58] Wolpert DM, Flanagan JR (2016). Computations underlying sensorimotor learning. Current Opinion in Neurobiology.

[59] Csikszentmihalyi, M. In *The Hidden Costs of Reward. New Perspectives on the Psychology of Human Motivation*, 205–216 (1978).

[60] Galea JM, Mallia E, Rothwell J, Diedrichsen J (2015). The dissociable effects of punishment and reward on motor learning. Nature Neuroscience.

[61] Mawase F, Uehara S, Bastian AJ, Celnik P (2017). Motor Learning Enhances Use-Dependent Plasticity. The Journal of Neuroscience.

[62] Diedrichsen J, White O, Newman D, Lally N (2010). Use-Dependent and Error-Based Learning of Motor Behaviors. Journal of Neuroscience.

[63] Huang VS, Haith AM, Mazzoni P, Krakauer JW (2011). Rethinking Motor Learning and Savings in Adaptation Paradigms: Model-Free Memory for Successful Actions Combines with Internal Models. Neuron.

[64] Taylor JA, Ivry RB (2014). Cerebellar and prefrontal cortex contributions to adaptation, strategies, and reinforcement learning. Progress in Brain Research.

[65] Brainard DH (1997). The Psychophysics Toolbox. Spatial Vision.

[66] Pelli DG (1997). The VideoToolbox software for visual psychophysics: transforming numbers into movies. Spatial Vision.

[67] Kleiner, M., Brainard, D.H. & Pelli, D.G. In *European Conference on Visual Perception* (2007).

