# Supplementary material for: Interacting Learning Processes during Skill Acquisition: Learning to control with gradually changing system dynamics
Source: Sci Rep. 2017 Oct 16;7:13191. doi: 10.1038/s41598-017-13510-0 (PMC5643438; doi:10.1038/s41598-017-13510-0)
Supplement: Supplementary file 1 — Supplementary Material 1 [file 41598_2017_13510_MOESM1_ESM.pdf]

Supplementary Material 1: Interacting Learning Processes during Skill Acquisition: Learning to control with gradually changing system dynamics

Nicolas Ludolph[1, 2*], Martin A. Giese[1], Winfried Ilg[1]

[1]Section Computational Sensomotorics, Department of Cognitive Neurology,
Hertie Institute for Clinical Brain Research, and Centre for Integrative Neuroscience, University of Tübingen, Germany
[2]International Max-Planck Research School for Cognitive and Systems Neuroscience, University of Tübingen, Germany

Corresponding author: Nicolas Ludolph
Department of Cognitive Neurology,
Hertie Institute for Clinical Brain Research,
Centre for Integrative Neuroscience
Otfried-Müller-Straße 25, 72076 Tübingen, Germany
Phone: +49 7071 29 89131
Email: nicolas.ludolph@uni-tuebingen.de

S1 APPENDIX

Numeric reward

Equation 1 describes the instantaneous reward, where s_x and s_θ denote the state variables for the cart position and pole angle and F denotes the applied force. These variables were normalized using the constraints to the range $[0, 1]$ by dividing by the maximum position (5m), pole angle (60°) or force (4N) respectively. Multiplication with the time-discretization constant Δt makes sure that the reward provided per second does not depend on the discretization, i.e. $r(s_x, s_\theta, F)$ describes the reward per time-discretization step. The remaining terms keep the reward per second between 0 and 10. The reward function takes the maximum value if the cart is in the centre ($s_x = 0$), the pole is vertical ($s_\theta = 0$) and no force ($F = 0$) is applied.

$$r(s_x, s_\theta, F) = \frac{10}{3}\left(3 - \frac{|s_x|}{5} - \frac{|s_\theta|}{60} - \frac{|F|}{4}\right)\Delta t \quad (1)$$

Reward per second

The cumulative reward was continuously updated and provided throughout each trial (number in the cart, see **Fig1** A in the main text), allowing subjects to infer the current reward per second and to improve further after being confident in balancing. Additionally, subjects were explicitly aware of the factors influencing the received reward per second. Thus, we analysed the average reward per second (**S1 Appendix Fig 1**) in order to examine whether subjects tried to optimize the provided numeric reward. We found that subjects in condition GG received more reward per second at the beginning (first 5 minutes, Wilcoxon rank sum, **$p<0.001$, median: GG=8.19, CG=7.77**) but approached, towards the end of the experiment, about the same amount of reward as subjects in condition CG (last 5 minutes, Wilcoxon rank sum, **$p=0.81$, median: GG=7.89, CG=7.95**). Regression analysis using a linear mixed-effects model was conducted to examine the effect of group and time on the reward per second. Although both factors and their interaction reached significance (all **$p<0.001$**), the reward per second did not change significantly over time (**$p=0.09$**) for subjects in condition CG, indicating that the numeric reward signal is not used by the subjects to optimize behaviour. Fitting the model to the last 45 minutes did not reveal any significant factors (all **$p>0.1$**). Hence, the initially higher reward per second observed for subjects in condition GG is potentially an artefact of slower state changes due to the low gravity.

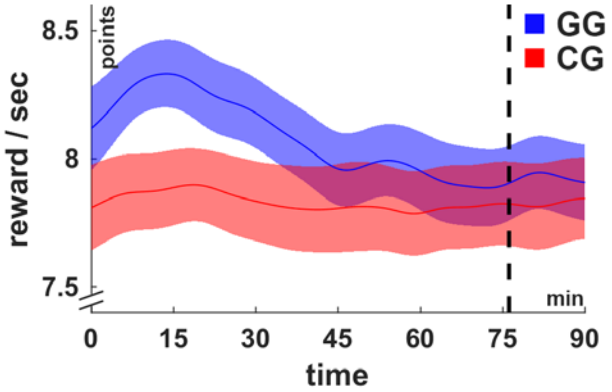

S1 Appendix Fig 1. Average course of reward per second over the course of the experiment. The reward per second ranges from zero to ten points. Even though the average reward per second is slightly higher in condition GG, the difference reaches significance only at the very beginning of the experiment. The shaded areas indicate the inter-subject-variability (±1 SEM). The black dashed line indicates the time at which all subjects in condition GG have reached maximum gravity (g_{max}=3.5m/s^2) latest. For the purpose of illustration, the curves were smoothed over time.

S1 FIGURE

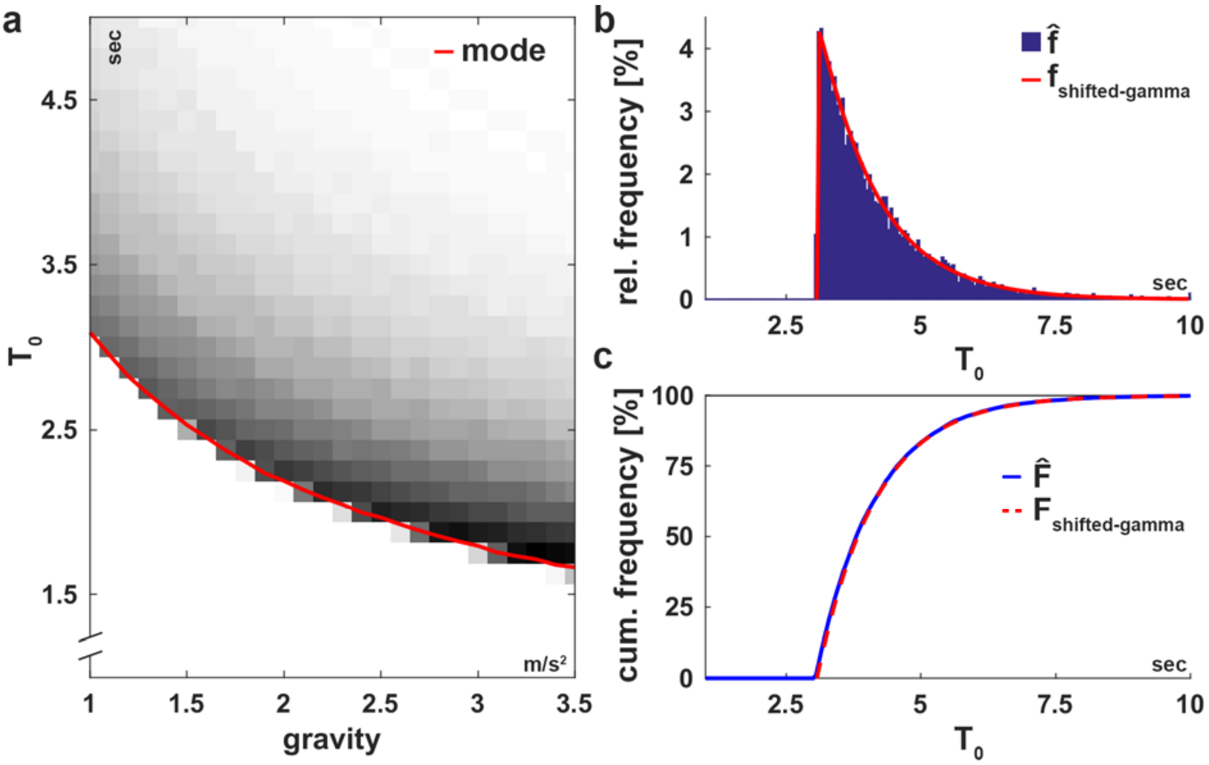

S1 Fig 2. The time T_0 as function of the gravity. (A) The time T_0 as function of the gravity is the trial length in the case that no input force is applied to the cart. The distribution of T_0 been determined by simulating the cart-pole system for each gravity step 1000 times with random initial pole angle. Each simulation yields one sample of T_0. The red line indicates the mode of fitted shifted-gamma distributions (see B). (B, C) The distribution of T_0 can be well described by a shifted gamma distribution (here exemplified for g_0=1.0m/s^2). (B) Empirical histogram $\hat{f}$ and (C) cumulative histogram $\hat{F}$ of the observed T_0's in comparison to the theoretical probability density function f and cumulative density function F of the fitted shifted-gamma distribution.

S2 FIGURE

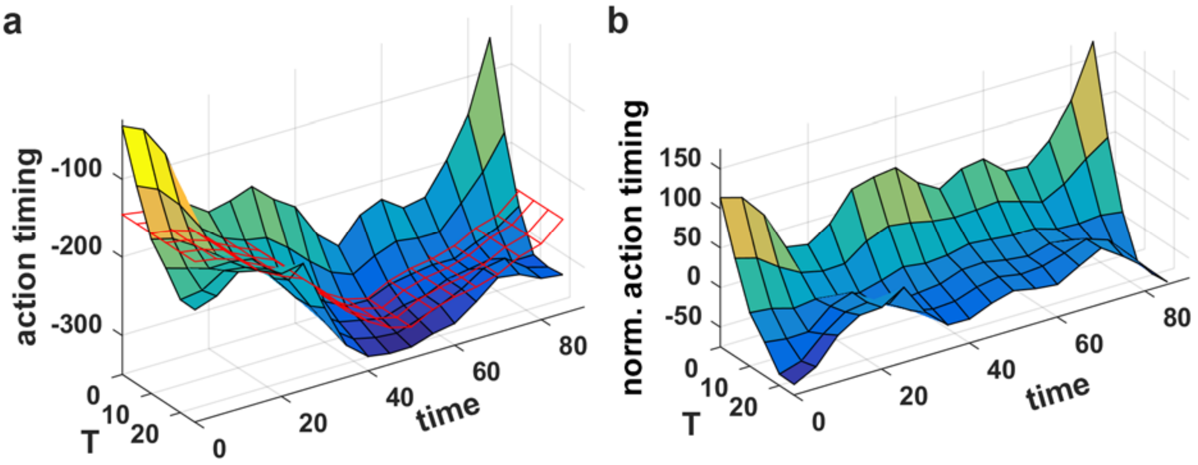

S2 Fig 3. Removing the influence of learning on the relation between the action timing and trial length. (A) Action timing as function of the trial length (T) and time in the experiment before normalization (coloured surface). The average action timing across all trial length bins within each time bin is illustrated as red mesh. (B) Subtraction cancels out the influence of learning on the relation between the action timing and trial length. Averaging across time bins yields Figure 6a of the main text. For the purpose of illustration, the surfaces were smoothed.
