# Supplementary material for: Interacting Learning Processes during Skill Acquisition: Learning to control with gradually changing system dynamics
Source: Sci Rep. 2017 Oct 16;7:13191. doi: 10.1038/s41598-017-13510-0 (PMC5643438; doi:10.1038/s41598-017-13510-0)
Supplement: Supplementary file 2 — Supplementary Material 2 [file 41598_2017_13510_MOESM2_ESM.pdf]

Supplementary Material 2:
Interacting Learning Processes during Skill Acquisition: Learning to control with gradually changing system dynamics

Nicolas Ludolph[1, 2*], Martin A. Giese[1], Winfried Ilg[1]

[1]Section Computational Sensomotorics, Department of Cognitive Neurology,
Hertie Institute for Clinical Brain Research, and Centre for Integrative Neuroscience, University of Tübingen, Germany
[2]International Max-Planck Research School for Cognitive and Systems Neuroscience, University of Tübingen, Germany

Corresponding author: Nicolas Ludolph
Department of Cognitive Neurology,
Hertie Institute for Clinical Brain Research,
Centre for Integrative Neuroscience
Otfried-Müller-Straße 25, 72076 Tübingen, Germany
Phone: +49 7071 29 89131
Email: nicolas.ludolph@uni-tuebingen.de

Dataset Description

In all *.csv files:

Subject	Subject ID
Group	Group of Subject

Dataset1.csv:

Age	Age of Subject
Gender	Gender of Subject
Tinit	Intitial avg. trial length (average over the first 5 minutes)
Tend	Final avg. trial length (average over the last 5 minutes)
TT0init	Intitial avg. normalized trial length (average over the first 5 minutes)
TT0end	Final avg. normalized trial length (average over the last 5 minutes)
IRinit	Initial avg. reward per second (average over the first 5 minutes)
IRend	Final avg. reward per second (average over the last 5 minutes)
SUCCfirst	Time till the first successful trial (minutes)
ISIm10	Avg. inter-success-interval over the first 10 successes

Dataset2.csv:

Pgw1	avg. (g: 1.0 - 3.5) trial length difference within gravity steps
Pga1	avg. (g: 1.0 - 3.5) trial length difference across gravity steps
ATgw1	avg. (g: 1.0 - 3.5) action timing difference within gravity steps
ATga1	avg. (g: 1.0 - 3.5) action timing difference across gravity steps
AVgw1	avg. (g: 1.0 - 3.5) action variability difference within gravity steps
AVga1	avg. (g: 1.0 - 3.5) action variability difference across gravity steps
Pgw2	avg. (g: 1.3 - 3.5) trial length difference within gravity steps
Pga2	avg. (g: 1.3 - 3.5) trial length difference across gravity steps
ATgw2	avg. (g: 1.3 - 3.5) action timing difference within gravity steps
ATga2	avg. (g: 1.3 - 3.5) action timing difference across gravity steps
AVgw2	avg. (g: 1.3 - 3.5) action variability difference within gravity steps
AVga2	avg. (g: 1.3 - 3.5) action variability difference across gravity steps

Dataset3.csv:

SuccessNr	Number of success
ISI	inter-success interval

Dataset4.csv:

Time	time passed since the beginning of the experiment
G	gravitational constant
T	trial length
TT0	normalized trial length
R	cumulated reward
IR	instantaneous reward

Dataset5.csv:

Time	time passed since the beginning of the experiment
AT	action timing
AV	action variability

Dataset6.csv:

Tbin	trial length bin
ATnl	normalized action timing
AVnl	normalized action variability
